# Seroprevalence of Antibodies against Measles, Rubella and Varicella among Asylum Seekers Arriving in Lower Saxony, Germany, November 2014–October 2015

**DOI:** 10.3390/ijerph13070650

**Published:** 2016-06-30

**Authors:** Salla E. Toikkanen, Armin Baillot, Johannes Dreesman, Elke Mertens

**Affiliations:** 1 Governmental Institute of Public Health of Lower Saxony (Niedersächsisches Landesgesundheitsamt, NLGA), Roesebeckstraße 4-6, Hanover 30449, Germany; Armin.Baillot@nlga.Niedersachsen.de (A.B.); Johannes.Dreesman@nlga.Niedersachsen.de (J.D.); Elke.Mertens@nlga.Niedersachsen.de (E.M.); 2 European Programme for Intervention Epidemiology Training (EPIET), European Centre for Disease Prevention and Control (ECDC), Tomtebodavägen 11a, Stockholm 17183, Sweden

**Keywords:** measles, rubella, varicella, asylum seekers, seroprevalence, IgG, immunisation

## Abstract

The number of asylum seekers arriving in Germany has increased rapidly since 2014 and cases of vaccine-preventable diseases at reception centres were reported. Asylum seekers 12 years and older arriving in Lower Saxony were serologically screened for antibodies against measles, rubella and varicella between November 2014 and October 2015. We calculated the seroprevalence from the screening data by disease, country of origin and age group and compared them to literature-based herd immunity thresholds in order to identify immunisation gaps. In total, 23,647 specimens were included in our study. Although the vast majority of asylum seekers tested positive for antibodies against measles, rubella and varicella, the seroprevalences were not sufficient to ensure herd immunity. The seroprevalences varied substantially between countries of origin and increased with age. Immunisation of asylum seekers against measles, rubella and varicella is needed and the detailed information on seroprevalences among subgroups of asylum seekers can be used for targeted immunisations at reception centres.

## 1. Introduction

The number of arriving asylum seekers in Germany reached new heights in 2015 when over 476,000 applications for asylum were registered—more than double the number in the previous year [1]. For a substantial number of arrived asylum seekers the application process was still pending at the time of the publication of these statistics and it was estimated that in reality more than 1 million asylum seekers arrived in Germany in 2015 [2].

Outbreaks of measles and varicella linked to asylum seekers were reported from all of Germany. A study conducted by the Robert Koch Institute (RKI) estimated that approximately one third of the 78 notified outbreaks in German reception centres between 2003 and 2013 were caused by either varicella or measles [3]. Between October 2014 and August 2015 a large measles outbreak with more than 1300 cases took place in Berlin, also affecting the asylum seekers population [4]. Just between calendar weeks 40 and 50/2015, 554 cases of varicella and four cases of measles among asylum seekers were reported to RKI [5].

In Germany, refugees are allocated to the federal states following the annually calculated quotas determined by the so called “Königsteiner Key” which is based on the federal states’ tax incomes and population. In 2015 the quota for Lower Saxony was 9.4%, yielding approximately over 100,000 arrived asylum seekers [6].

According to German Law, asylum seekers are required to first be located in reception centres and undergo a medical examination of which extent is decided at the federal state level. In Lower Saxony, the Federal Regulation specified the examination to include serological screening of antibodies against measles, rubella, and varicella for all arriving persons 12 years and older between November 2014 and October 2015 [7].

At that time, a comprehensive vaccination strategy for asylum seekers in reception centres was not implemented and the main purpose of the screening was to aid the centres in outbreak management, i.e., targeting the outbreak control measures such as isolation, suspending relocations, and vaccinations. We analysed the serological data available in order to detect immunisation gaps in subpopulations of asylum seekers and evaluate the need for vaccination.

## 2. Materials and Methods

### 2.1. Microbiological Analysis

Blood samples from asylum seekers together with demographic information including name, date of birth, sex and country of origin, were sent to a microbiological laboratory for testing. In Germany, the ligand binding assays (e.g., enzyme immunoassays) are recommended for the measles and rubella IgG antibody detection by the societies of virology (Gesellschaft für Virologie e.V., Ulm, Germany and Deutsche Vereinigung zur Bekämpfung der Viruskrankheiten e.V., Homburg, Germany). Serum samples sent to the Governmental Institute of Public Health of Lower Saxony (NLGA) were tested by measles and varicella zoster virus-ELISA IgG (Sukisui Virotech GmbH, Rüsselsheim, Germany) and rubella virus SERION ELISA classic IgG (Virion/Serion GmbH, Würzburg, Germany) for the presence of IgG antibodies and stored at −20 °C. The NLGA laboratory is accredited according to DIN EN ISO 15189 including an extensive quality management system. A qualitative method was used for detecting the presence of measles and varicella antibodies and a quantitative method for the detection of rubella antibodies. The sensitivities of the assays were 98.6% for measles and 99.0% for varicella and the specificities >99.8% for measles and 94.0% for varicella according to manufacturer’s report. For rubella, the seropositivity was derived from the quantitative result as having anti-rubella antibody concentration of >20 IU/mL according to the manufacturer’s guidelines. The manufacturer reported the sensitivity and specificity of the test being 99.1% and >98.9%, respectively. The results from serum samples analysed in microbiological laboratories other than NLGA were not available.

### 2.2. Statistical Analysis

Seroprevalence was calculated as the number of positive test results divided by the number of performed tests together with the 95% exact Clopper-Pearson confidence intervals (95% CI). The seroprevalence was determined by disease, country of origin, sex and three age groups: 12–29 years, 30–44 years and 45 years and older. The differences in seroprevalences between sexes were compared and the statistical significances assessed with chi-squared test. After stratification by age groups, the linear change over age in the seroprevalence was tested with chi-squared test for trend in proportions.

The obtained seroprevalences were additionally compared with literature based thresholds; for measles and rubella this threshold was 95% according to the WHO measles and rubella elimination goals [8,9,10] and for varicella a herd immunity threshold of 91% was used [11]. If the seroprevalence in a particular group exceeded statistically significantly the corresponding threshold, the group was defined to be sufficiently protected in the sense of having achieved an adequately low level of susceptibility for the infection. One-tailed Z-score test was used to determine the statistical significance.

Finally, logistic regression models were fitted to examine whether sex or age were associated with having antibodies against measles, rubella and varicella after adjustment for the country of origin. The dichotomous seropositivity status (1 = Yes, 0 = No/borderline) was treated as dependent variable and sex and country of origin were entered into the models as independent categorical variables together with the age as continuous variable. *p*-values < 0.05 were considered statistically significant. The results obtained from strata with less than 30 asylum seekers are not presented in the tables or figures due to the large margins of error. All analyses were performed using R software, version 3.2.4 (R Core Team, Vienna, Austria) [12].

## 3. Results

### 3.1. Descriptive Statistics

Overall, 26,543 specimens were sent to NLGA between November 2014 and October 2015. Altogether, 2896 (10.9%) of the samples were excluded from the study due to missing information on age or origin or the test could not be performed due to poor sample or documentation quality yielding a total of 23,647 study specimens.

The median age of the screened asylum seekers was 26 years (range 12–94 years, IQR = 33 − 21 years). 63.9% (15,121) belonged to age group 12–29 years, 27.8% (6567) to 30–44 years and 8.3% (1959) were 45 years or older. Of 21,059 specimens sent with information on sex, 75.6% (15,928) were from males. For 2588 (10.9%) the information on sex was missing. In general, females were slightly older than males: the median age among females was 28 years (range 12–86 years) compared to 25 years (range 12–85 years) among males.

For 66 individuals, the origin was recorded with continent or sub-continent level but without exact country of origin information: Southern Africa was indicated for one person, Western Europe for three, Africa for two and Asia for 60 persons. These individuals were excluded from the stratified analysis by country of origin. Following the United Nations geoscheme [13], 37.4% (8823) of the 23,585 tested individuals with at least sub-continental origin information originated from Western Asia, 26.1% (6165) from Southern Europe and 15.4% (3627) from Northern Africa. The most frequently reported countries of origin were the Syrian Arab Republic (5135/23,581, 21.8%), Iraq (2822, 11.9%) and Montenegro (2525, 10.7%). Table 1 summarizes the ten most frequently reported countries of origin. In total, 62 countries of origin were reported with 27 of them reported at least 30 times.

The monthly number of tested samples increased from around 1500 samples between November 2014 and June 2015 to over 4000 samples in September–November 2015. This was mainly due to the increase in number of asylum seekers from Western Asia: 114 specimens from asylum seekers originating from the Syrian Arab Republic were analysed on average per month between November 2014 and June 2015 compared to the monthly average of 1319 specimens during the last three months of the study period.

### 3.2. Seroprevalence

In total, test results from 23,647 samples were available for measles and varicella and 23,643 for rubella. The overall measles seroprevalence was 79.9% with a total of 18,896 positive test results. The rubella and varicella seroprevalences were 85.1% (20,132/23,643) and 87.5% (20,693/23,647), respectively (Table 2). None of these seroprevalences exceeded the defined herd immunity thresholds statistically significantly. A total of 1264/23,647 (5.3%) of the measles, 2015/23,643 (8.5%) of the rubella and 678/23,647 (2.9%) of the varicella test results were classified borderline.

#### 3.2.1. Sex and Age Groups

Overall, males were better protected against measles and rubella than females (*p*-value < 0.001 for both). For varicella, the situation was reversed: a higher proportion of females had the antibodies compared to males (*p*-value < 0.001) (Table 2).

When stratified only by age groups, the seroprevalence increased statistically significantly with age for all antibodies (*p*-values < 0.001) and overall, i.e., irrespective of the sex or country of origin, the asylum seekers 45 years and older were sufficiently protected against measles and varicella (*p*-value < 0.001 for varicella, 0.006 for measles). The measles seroprevalence increased from 74.9% in the youngest age group to 96.3% in the oldest age group. For rubella, these figures were 83.3% for the youngest and 88.3% for the oldest age group and for varicella 84.9% and 94.3% respectively (Table 3).

#### 3.2.2. Countries of Origin

**Figure 1:**
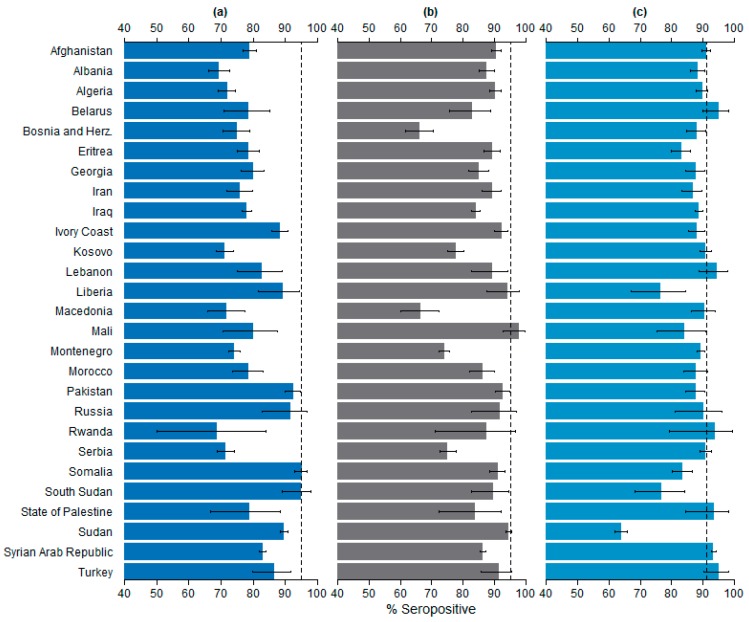
Seroprevalence by country of origin with 95% confidence intervals: (**a**) Measles; (**b**) Rubella; (**c**) Varicella. The dotted lines mark the herd immunity thresholds (95% for measles and rubella, 91% for varicella [8,9,10,11]).

The seroprevalences stratified by country of origin are presented in Figure 1.

##### Measles

None of the groups were sufficiently protected against measles. The highest seroprevalence was measured among asylum seekers from Somalia, 95.2% (513/539, 95% CI 93.0%–96.8%) and the lowest 68.8% among the asylum seekers from Rwanda (22/32, 95% CI 50.0%–83.9%). All Western Balkan countries and Algeria had seroprevalences below 75%. Over 90% seroprevalences were measured among South Sudanese (111/117, 94.9%, 95% CI 89.2%–98.1%), Pakistani (475/513, 92.6%, 95% CI 90.0%–94.7%) and Russian (66/72, 91.7%, 95% CI 82.7%–96.9%) asylum seekers.

##### Rubella

Asylum seekers from Mali had the highest rubella seroprevalence (93/95, 97.9%, 95% CI 92.6%–99.7%). Also asylum seekers originating from Sudan (2078/2196, 94.6%, 95% CI 93.6%–95.5%), Liberia (96/102, 94.1%, 95% CI 87.6%–97.8%), Pakistan (476/513, 92.8%, 95% CI 90.2%–94.9%), Ivory Coast (584/632, 92.4%, 95% CI 90.1%–94.3%), Russia (66/72, 91.7%, 95% CI 82.7%–96.9%), Turkey (130/142, 91.5%, 95% CI 85.7%–95.6%), Somalia (491/539, 91.1%, 95% CI 88.4%–93.4%), Afghanistan (1390/1535, 90.6%, 95% CI 89.0%–92.0%) and Algeria (1000/1107, 90.3%, 95% CI 88.4%–92.0%) had a seroprevalence above 90%. None of the groups had a seroprevalence statistically significantly over the 95% threshold. The Western Balkan countries, excluding Albania, had the lowest seroprevalences: all below 80%, the lowest being Bosnia and Herzegovina (309/467, 66.2%, 95% CI 61.7%–70.5%).

##### Varicella

One group was sufficiently protected against varicella, that being asylum seekers originating from the Syrian Arab Republic with the seroprevalence of 93.4% (4794/5135, 95% CI 92.6%–94.0%, *p*-value < 0.001). Sudanese asylum seekers had the lowest seroprevalence: 64.0% (1405/2196, 95% CI 61.9%–66.0%).

#### 3.2.3. Countries of Origin by Age Groups

**Figure 2:**
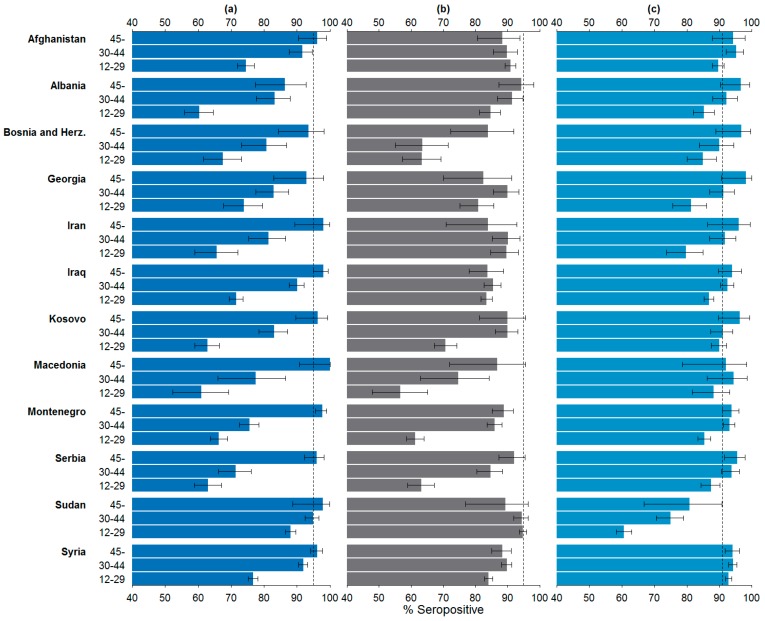
Seroprevalence by country of origin and age group (12–29 years, 30–44 years, 45 years and older) with 95% confidence intervals: (**a**) Measles; (**b**) Rubella; (**c**) Varicella. The dotted lines mark the herd immunity thresholds (95% for measles and rubella, 91% for varicella [8,9,10,11]).

The stratification by age groups was performed for twelve countries of origin with at least 30 asylum seekers in each age group. The seroprevalences by country of origin and age group are visualised in Figure 2.

##### Measles

The stratification by age groups showed that asylum seekers in the age group 45 years and older originating from Iraq (194/198, 98.0%, 95% CI 94.9%–99.4%, *p*-value 0.039) and Montenegro (376/385, 97.7%, 95% CI 95.6%–98.9%, *p*-value 0.011) were sufficiently protected against measles. Under 65% seroprevalence were observed in the youngest age group among asylum seekers from Albania (296/491, 60.3%, 95% CI 55.8%–64.6%), Macedonia (83/136, 61.0%, 95% CI 52.3%–69.3%), Kosovo (415/661, 62.8%, 95% CI 59.0%–66.5%) and Serbia (345/548, 63.0%, 95% CI 58.8%–67.0%).

##### Rubella

For rubella, the stratification did not reveal any age groups with sufficient protection. The seroprevalence was lowest in the youngest age group among asylum seekers from Macedonia (77/136, 56.6%, 95% CI 47.9%–65.1%), Montenegro (774/1264, 61.2%, 95% CI 58.5%–63.9%) and Serbia (346/548, 63.1%, 95% CI 58.9%–67.2%).

##### Varicella

Against varicella, all age groups of asylum seekers from Syrian Arab Republic were sufficiently protected (age group 12–29 years: 2886/3109, 92.8%, 95% CI 91.9%–93.7%, *p*-value < 0.001; 30–44 years: 1456/1546, 94.2%, 95% CI 92.9%–95.3%, *p*-value < 0.001; 45– years: 452/480, 94.2%, 95% CI 91.7%–96.1%, *p*-value 0.010) as well as asylum seekers aged 30 and above from Montenegro (30–44 years: 816/876, 93.2%, 95% CI 91.3%–64.7%, *p*-value 0.015; 45– years: 361/385, 93.8%, 95% CI 90.9%–96.0%, *p*-value 0.035), Serbia (30–44 years: 314/335, 93.7%, 95% CI 90.6%–96.1%, *p*-value 0.049; 45– years: 191/200, 95.5%, 95% CI 91.6%–97.9%, *p*-value 0.018) and Afghan asylum seekers between 30 and 44 years (261/27, 95.3%, 95% CI 92.0%–97.4%, *p*-value 0.009). The lowest seroprevalence was among Sudanese asylum seekers younger than 45 years (12–29 years: 1032/1702, 60.6%, 95% CI 58.3%–63.0%; 30–44 years: 335/447, 74.9%, 95% CI 70.7%–78.9%). Also individuals from Iran between 12 and 29 years had a seroprevalence below 80% (170/213, 79.8%, 95% CI 73.8%–85.0%).

#### 3.2.4. Factors Associated with Seropositivity

A multivariable logistic regression model was fitted separately for measles, rubella and varicella. The results are summarized in Table 4.

Sex did not have a statistically significant effect on the seropositivity for measles after adjustment with the country of origin and age (*p*-value 0.81). Age, however, did have a positive effect on the seropositivity for measles: one year increase in age increased the odds of being seropositive with 6% (*p*-value < 0.001) after taking the country of origin and sex into account. For rubella, sex did have an effect on the seropositivity even after adjustment for age and country of origin—the male odds ratio was 1.32 times higher compared to female (*p*-value < 0.001). Also age had a positive effect on the seropositivity for rubella (*p*-value <0.001). For varicella, sex did not have a statistically significant association with being seropositive after the adjustment (*p*-value 0.590) but one year increase in age increased the odds for being seropositive with 4% (*p*-value < 0.001) (Table 4).

## 4. Discussion 

We performed an extensive serological screening of antibodies against measles, rubella and varicella. Prior seroprevalence data among asylum seekers was scarce. Serological screening results from the U.S. for refugee children and adolescents were published in 2002 [14], but to our knowledge no recent data from Europe has been published.

Although the majority of asylum seekers in Lower Saxony tested positive for antibodies against measles, rubella and varicella, the level of immunity was not sufficient to achieve a desirable level of susceptibility among asylum seekers in the reception centres. Stratification by country of origin showed that the seroprevalence varied between countries of origin, but very few subgroups of asylum seekers achieved the immunity thresholds. The differences in seroprevalences between countries of origin could be due to differences in endemicity and access to health care and varying immunisation strategies. In general, the seroprevalences of measles, rubella and varicella antibodies increased with age putting the young adults and adolescents at a higher risk of infection.

Moreover, sex had an effect on the rubella seropositivity, even after adjustment for age and country of origin. Females were less likely to have protective antibodies against rubella. This finding is surprising and needs further verification. Perhaps female asylum seekers travel to Lower Saxony more directly from their countries of origin than males, for example because of family reunification, and thus avoid possible immunisation activities that take place on transit camps. This could also reflect persisting gender inequality on the access to the health care in the countries of origin.

No information on children younger than 12 years of age was available and interpolating the results to this age group was thus not possible. In Lower Saxony, the serological screenings were implemented only on persons 12 years and older due to practical reasons: drawing a blood sample is considerably easier from adults and older children than from young children. Moreover, the majority of the arriving asylum seekers in Lower Saxony were young adults below 30 years of age rather than children. There was also a reason to assume that the medical workers in reception centres already prioritised the immunisation activities on children due to the existing recommendations from the German Standing Committee on Vaccination (STIKO) on the primary immunisation of children between 11 and 23 months of age against measles, rubella and varicella with a catch-up among unvaccinated or insufficiently vaccinated persons younger than 18 years of age against measles and rubella [15].

The number of blood specimens analysed outside NLGA as well as the number of laboratories where the reception centres sent samples for analysis is unknown. However, the reception centres were instructed to send the serum samples to NLGA and thus the number of specimens analysed elsewhere was presumably low. Despite the Federal Regulations for the medical examinations of the asylum seekers, it is very likely that the blood specimens were not systematically obtained from all asylum seekers in the target age group due to lack of medical resources in reception centres during the temporal peaks of arriving individuals. We believe, however, that this does not introduce selection bias to our estimates and that our data provides a representative overview of the situation among asylum seekers in Lower Saxony during the study period.

In our study, we have used herd immunity thresholds derived from literature to quantify the level of protection against measles, rubella and varicella. The herd immunity thresholds provide a theoretical framework for the risk assessment of person-to-person transmission of an infectious disease: when the prevalence of protected individuals is higher than the corresponding threshold, the transmission is unlikely and the risk of disease outbreaks is minimised. The required prevalence of protected individuals depends also on the population characteristics and different sources suggest different herd immunity thresholds [16]. Our choice of the used thresholds was based on the WHO targets for measles and rubella eradication [8,9,10] and the more conservative threshold of 91% given in a paper by Plans-Rubió for varicella [11]. However, in a reception centre setting where overcrowding may occur, the contact rate between persons is high and arriving asylum seekers suffer physically from their journey and thus might be more susceptible to infections, it can be argued that even these relatively high thresholds may be too low to obtain outbreak-free circumstances. Nevertheless, a comparison to these thresholds serves as a good starting point in identifying groups who would benefit the most from the immunisation. Achieving these goals within different asylum seeker subpopulations would aid in maintaining high level of protection in the reception centres.

Before the mass influx of asylum seekers in autumn 2014, there were three operating reception centres with the initial capacity of around 600 persons each in Lower Saxony. Over the course of the refugee movement, the reception centres’ capacities were first expanded and during autumn 2015 a number of new reception centres were established up to a maximum of 75 centres with the total capacity of over 30,000 persons. The country of origin, age and sex distribution of the asylum seekers differed between the centres and also changed over time. The stratification by sex, age and country of origin provides more accurate information of the individual centres’ susceptibility profiles than the overall figures.

The borderline test results were included in the denominator in the calculations of the seroprevalences. This choice was justified by the attempt to provide the most conservative scenario for public health actions. Excluding the borderline results from the analysis would have yielded higher immunity estimates but would have provided an overly optimistic impression on the immunisation needs.

At the time of the serological screenings, a comprehensive vaccination strategy for asylum seekers was not implemented in the reception centres. Following the existing national STIKO recommendations, no emphasis was put on vaccinations that should be administered shortly after the arrival of asylum seeker. Instead, a complete set of vaccinations including basic immunisation against several pathogens was recommended for persons without immunisation history or documented vaccinations [15]. Thus the immunisation could be interpreted as a task which should rather be performed at the final location of the asylum seeker in order to guarantee the completeness. In October 2015, the Ministry of Social Affairs, Health and Equal Opportunities of Lower Saxony issued a specific recommendation for the vaccinations that should be prioritised in the reception centres including the trivalent measles-mumps-rubella (MMR) vaccination for all asylum seekers 13 years and older without documented immunisation [17]. This follows the RKI concept paper from October 2015 on the implementation of early vaccinations for asylum seekers arriving in Germany [18]. However, the immunisation strategies are not yet standardised across the reception centres and the implementation is limited due to strained resources.

In a measles outbreak in a German reception centre, the implementation of serological screening of the asylum seekers and the following selective immunisation based on the screening results was shown to be a cost-ineffective strategy compared to a mass-vaccination campaign [19]. However, the utilisation of the existing data on sub-groups of asylum seekers in prioritizing the vaccinations to the populations with lowest seroprevalence, as well as to the commonly known risk groups, can provide an efficient outbreak control and prevention strategy when mass vaccinations cannot be executed. In Lower Saxony, a targeted vaccination strategy based on available seroprevalence information has already been successfully used in varicella outbreak management in reception centres.

## 5. Conclusions

Only few subgroups of arriving asylum seekers in Lower Saxony were sufficiently protected against measles, rubella and varicella and the majority of adolescents and adults would benefit from immunisation activities. The immunisation strategies should be planned to comply with WHO’s measles and rubella elimination targets in the WHO European Region [11]. In situations when all arriving asylum seeker cannot be vaccinated against these infections, the utilisation of seroprevalence information on sub-groups of asylum seekers can aid the prioritisation of the measures to the most vulnerable groups. This has been proven to be successful in reception centres during varicella outbreaks in Lower Saxony.

## Figures and Tables

**Table 1:** Ten most frequently reported countries of origin among asylum seekers arriving in Lower Saxony, November 2014–October 2015.

Country of Origin (*N* = 23,581)	No.	%
Syrian Arab Republic	5135	21.8
Iraq	2822	12.0
Montenegro	2525	10.7
Sudan	2196	9.3
Afghanistan	1535	6.5
Algeria	1107	4.7
Serbia	1083	4.6
Kosovo	1045	4.4
Albania	799	3.4
Ivory Coast	632	2.7

**Table 2:** Number of positive and tested samples and seroprevalence with 95% confidence intervals for measles, rubella and varicella antibodies.

	Sex	No. Positive	No. Tested	% Positive (95% CI)
Measles	Male	12,880	15,928	80.9 (80.2–81.5)
	Female	4030	5131	78.5 (77.4–79.7)
	Total	18,896	23,647	79.9 (79.4–80.4)
Rubella	Male	13,907	15,926	87.3 (86.8–87.8)
	Female	4104	5129	80.0 (78.9–81.1)
	Total	20,132	23,643	85.1 (84.7–85.6)
Varicella	Male	13,828	15,928	86.8 (86.3–87.3)
	Female	4610	5131	89.8 (89.0–90.7)
	Total	20,693	23,647	87.5 (87.1–87.9)

**Table 3:** Number of positive and tested samples and seroprevalence with 95% confidence intervals for measles, rubella and varicella antibodies stratified by age group.

	Age Group	No. Positive	No. Tested	% Positive (95% CI)
Measles	12–29	11,328	15,121	74.9 (74.2–75.6)
	30–44	5682	6567	86.5 (85.7–87.3)
	45–	1886	1959	96.3 (95.3–97.1)
Rubella	12–29	12,600	15,119	83.3 (82.7–83.9)
	30–44	5803	6567	88.4 (87.6–89.1)
	45–	1729	1957	88.3 (86.8–89.7)
Varicella	12–29	12,835	15,121	84.9 (84.3–85.4)
	30–44	6011	6567	91.5 (90.8–92.2)
	45–	1847	1959	94.3 (93.2–95.3)

**Table 4:** Results from multivariable logistic regression models with the seropositivity status (Yes/No) as dependent variable and sex, age and country of origin as independent variables. The output for countries of origin is omitted.

	Factor		OR (95% CI)	Z-Statistic	*p*-Value
Measles	Sex	Female	(Ref.)		
		Male	1.01 (0.93–1.10)	0.24	0.813
	Age		1.06 (1.06–1.07)	27.7	<0.001
Rubella	Sex	Female	(Ref.)		
		Male	1.32 (1.20–1.44)	6.03	<0.001
	Age		1.03 (1.03–1.04)	15.2	<0.001
Varicella	Sex	Female	(Ref.)		
		Male	1.03 (0.92–1.15)	0.54	0.590
	Age		1.04 (1.03–1.04)	13.3	<0.001
